# Melanocytic tumors with spitzoid morphology: correlation of clinicopathologic features and FISH analysis

**DOI:** 10.55730/1300-0144.5656

**Published:** 2023-04-07

**Authors:** Deniz ÜNLÜER KAPIŞKAY, Övgü AYDIN ÜLGEN, Ümit İNCE, Cuyan DEMİRKESEN

**Affiliations:** 1 Department of Pathology, Republic of Türkiye Ministry of Health Bozüyük General Hospital, Bilecik, Turkiye; 2 Department of Pathology, Cerrahpaşa School of Medicine, İstanbul University-Cerrahpaşa, İstanbul, Turkiye; 3 Department of Pathology, School of Medicine, Acıbadem University, İstanbul, Turkiye

**Keywords:** Melanoma, atypical Spitz tumor, FISH, 9p21, RREB-1, CDK2NA

## Abstract

**Background/aim:**

A subset of melanocytic tumors with spitzoid morphology may lead to potential inaccurate diagnosis and lack of assessment of malignancy potential.

In this study, we aimed to evaluate melanocytic tumors with spitzoid morphology using conventional melanoma FISH (RREB-1, CCND1, MYB and CEP6) and 9p21 FISH (CDKN2A) probes and compare the probe results with clinical and histopathological features.

**Materials and methods:**

This study is a multicentric retrospective study including three centers, İstanbul University-Cerrahpaşa, Cerrahpaşa School of Medicine, Department of Pathology, Acıbadem University, School of Medicine, Department of Pathology and ETA Pathology Laboratory. The pathology reports in archives of these three centers between 2015 and 2017 have been reviewed for cases diagnosed as atypical Spitz tumor or melanoma with Spitzoid features. These cases were selected for the study. We analyzed 39 cases of atypical Spitz tumor (AST), 10 cases of melanomas with spitzoid features for clinicopathological data and chromosomal alterations, targeting RREB-1 (6p25), CCND1 (11q13), MYB (6q23), together with 9p21 (CDKN2A), using FISH methodology.

**Results:**

Thirty out of total 49 cases showed chromosomal alterations by 4-probe melanoma FISH assay, 22 (56.4%) cases were ASTs, and 8 (80%) cases were melanomas. Eighteen out of 49 cases showed homozygote deletion by 9p21 FISH assay, 12 (30.8%) cases were ASTs, and 6 (60%) cases were melanomas. When histopathological data were compared with FISH results, a statistically significant correlation was found between 9p21 FISH positivity (homozygous deletion) and presence of deep mitosis (p < 0.05). In addition, epidermal consumption (p = 0.07) and increased mitotic activity (p = 0.05) were more frequent in cases with homozygous 9p21 deletion, but these differences did not reach statistical significance. When the clinical features were considered, there was a statistically significant correlation between 9p21 FISH positivity and the diameter (p < 0.05). There was no statistically significant correlation between melanoma FISH assay and any of the histopathological or clinical data.

**Conclusion:**

These data suggest that 9p21 FISH positivity correlated with more worrisome histopathologic and clinical features, such as deep mitosis, increased mitotic activity, epidermal consumption, and larger lesion size, so these features are precious, pointing out spitzoid lesions with higher risk. However, there is a need for further studies using FISH or similar techniques in order to provide more accurate prognostic information in lesions Blank morphology.

## 1. Introduction

Spitz nevus was first described as “juvenile melanomas” due to their histopathological features similar to the conventional melanomas [1]. Later on, it has been suggested that these lesions may also have “borderline” and “malignant” forms [2, 3]. While some spitzoid lesions can metastasize to regional lymph nodes, clinical follow-up often does not reveal poor prognosis such as distant metastasis, recurrence, and disease related death, although rare cases with fatal outcome have been reported in the literature [4, 5].

Recently, it is discovered that melanocytic lesions with Spitz lineage have either kinase fusions or HRAS (Harvey Rat sarcoma virus-GTPase) mutation. However, sometimes melanomas may have spitzoid features without harboring the genomic changes. These tumors are called spitzoid melanomas and they have similar prognosis as conventional melanomas, differing from Spitz melanomas [6,7].

Although routine light microscopic examination remains the gold standard for diagnosis of Spitz tumors, confirmation is needed, using adjunctive methods, such as immunohistochemical and molecular techniques for assessment of malignancy potential and appropriate risk stratification. Methods such as comparative genomic hybridization (CGH), fluorescence in situ hybridization (FISH), next generation sequencing (NGS) and mutation analysis applied in the recent years, provides more detailed and accurate information regarding the molecular basis of these lesions.

Conventional melanoma FISH probes targeting RREB-1 (6p25) (ras responsive element binding protein 1), CCND1 (11q13) (cyclin D1), MYB(6q23) (myeloblastosis proto-oncogene) provide important diagnostic information in many melanocytic tumors, while their sensitivity and the specificity in spitzoid lesions are low, necessitating the development of newer probe designs [8].

Recent data suggest that the application of 9p21 FISH probe, which is based on the 9th chromosome deletions, in addition to the classical FISH probes, increased the sensitivity and specificity values for accurate diagnosis [9, 10].

In this study, we examined conventional melanoma FISH and 9p21 FISH probe positivity in a large series of tissue samples obtained from patients, that have melanocytic tumors with spitzoid features. We hypothesized that FISH probe positivity would be higher in samples obtained from patients with more worrisome histopathological features. In an exploratory analysis, we examined the correlation between FISH probe positivity and histopathological, clinical and genetic features of these spitzoid melanocytic lesions.

## 2. Materials and methods

This study is a multicentric retrospective study including three centers, İstanbul University-Cerrahpaşa, Cerrahpaşa School of Medicine, Department of Pathology, Acıbadem University, School of Medicine, Department of Pathology and ETA Pathology Laboratory. The pathology reports in archives of these three centers between 2015 and 2017 have been reviewed for cases diagnosed as atypical Spitz tumor or melanoma with Spitzoid features.

Forty-nine cases of melanocytic tumor, displaying spitzoid features, with adequate tissue in paraffin blocks for molecular studies were included in the study. Spitzoid histologic features can be listed as epidermal hyperplasia, hyperkeratosis, epithelioid morphology with amphophilic/glassy cytoplasm and a vesicular nucleus. Out of 49, 39 of the cases were previously diagnosed as atypical Spitz tumor (AST), whereas 10 were diagnosed as melanoma with spitzoid features (either Spitz melanoma or Spitzoid melanoma according to WHO, 2018 classification). According to the recent classification, cases of AST can be considered as “melanocytoma with spitzoid features”. We also included 2 Spitz nevi as negative controls and 2 nodular malignant melanomas as positive controls. Of note, Spitz melanomas, included in the group of the melanomas with spitzoid features in this analysis can at times be designated as “AST with high risk” by others; however, we selected to categorize them as melanomas for the purposes of this particular study since they met the sufficient histopathologic features for the diagnosis of melanoma [11].

### 2.1. Clinical and histopathological evaluation

The clinical and demographic data such as age, sex, localization, duration of the lesion and diameter were extracted from the patients’ pathology reports. Sentinel lymph node biopsy and regional lymph node dissection results were examined, if available. Additional clinical data were obtained for follow-up period, disease recurrence, lymph node involvement, and metastatic status for prognostic evaluation. The H&E (hematoxylin and eosin) sections of each case were reevaluated for accurate diagnosis by blinded pathologists. In the histopathological examination, symmetry in the primary tumor, presence of maturation, subcutaneous fat tissue involvement, ulceration, epidermal consumption, intraepidermal pagetoid spread, presence of Kamino bodies, cytological atypia and pleomorphism, presence of expansile nodule, mitosis (number and location), atypical mitosis, and lymphocytic infiltration were examined. Breslow thickness was noted for each case.

### 2.2. FISH evaluation

In the sections of blocks with adequate tumor cells (at least 60 tumor cells), areas with the highest tumor cell density and the least amount of pigment were marked. FISH technique was performed on 4 μm-thick slides, and RREB-1 gene amplification, CCND1 gene amplification, MYB gene deletion and 9p21 homozygous-heterozygous deletions were investigated using FISH commercial probes (For 9p21; 9p21 Vysis LSI p16 Spectrum Orange, Abbott Molecular and CEP9 Vysis CEP9 Spectrum Green, Abbott Molecular, for conventional melanoma FISH panel; CCND1 (11q13) Spectrum Green, MYB (6q23) Spectrum Gold, RREB-1 (6p25) Red Empire Genomics, Buffalo NY and D6Z1 (CEP6) Spectrum Aqua Abbott Molecular, Des Plains IL).

For the evaluation of conventional melanoma FISH and 9p21 FISH panels, regions with the highest tumor cell density and clearly seen tumor cell nuclei were selected in the images transferred to the computer, and at least 60 tumor cell nuclei were counted. The DAPI filter has been used to select cell nuclei clearly, and to use regions with minimal artifacts. Counts were made blind, without knowing the original diagnosis. The UCSF criteria were used for the evaluation [12].

Cells that have 2 green and 2 orange signals were considered to have normal FISH profiles when the 9p21 gene profile was evaluated. If there were no orange signals but 2 or more green signals in a cell, the cell was considered to have homozygous deletion. Cells with more than 2, but equal amounts of green and orange signals were noted as cells with normal FISH profile but polyploidy. In cases where the distance between two signals is smaller than the size at which 1 signal can fit, these signals were considered as a single signal. Homozygous deletion detected at a rate greater than 29% was considered FISH-positive for 9p21 [10].

### 2.3. Statistical analysis

The cases we used in our study are divided into 2 groups according to their diagnosis: atypical spitzoid tumors (n = 39) and spitzoid malignant melanomas (n = 10). Mean, standard deviation, median, minimum, and maximum values for continuous variables were provided as descriptive statistics. Numerical and percentage values were calculated for intermittent variables. Kolmogorov-Smirnov and Shapiro-Wilk tests were used for the evaluation of normal distribution in parameters such as age, sex, lesion duration, localization and histopathological features for these groups. Later, the groups were reclassified as classical melanoma FISH positive and negative cases, 9p21 FISH positive and negative cases, and both classical melanoma FISH and 9p21 FISH positive and negative cases according to the FISH analyzes. In these groups, the results of the FISH analyzes were compared with the clinical and histopathological features of the cases. Student’s t-test was used for parametric data and Mann-Whitney U, Chi-square and Fisher’s exact tests were used for nonparametric data in comparisons between groups. The Kappa test was used to assess intertest compliance. Kappa values are classified as low compliance at 00.21–00.40, moderate compliance at 0.41–0.60, good compliance at 0.61–0.80, and excellent compliance at 0.81–1.00 [13]. The results were evaluated with 95% confidence interval and statistical significance was defined as p < 0.05. SPSS version 21.0 (Statistical Package for the Social Sciences, IBM Corporation, Armonk, NY, USA) was used for statistical analysis.

## 3. Results

### 3.1. Clinical features

**Figure 1:**
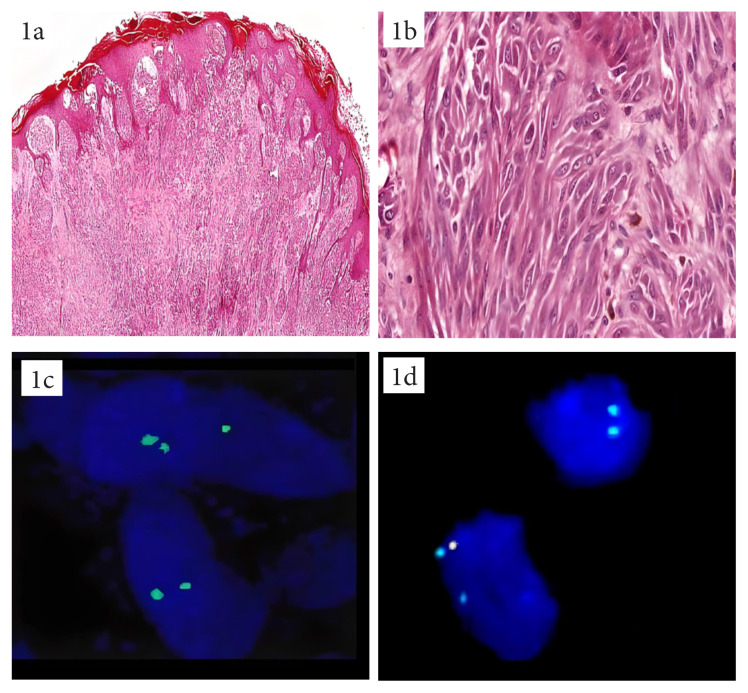
SMM, a 41-year-old patient with regional lymph node involvement (HEx40). Atypical melanocytes with spitzoid cytological features (HEX400) (1a, 1b), 9p21 FISH-positive (cells showing 9p21 homozygous deletion) (1c), melanoma FISH-positive (isolated MYB gene deletion) (1d).

The clinical features and the postdiagnostic parameters such as the status of sentinel lymph node biopsy, regional lymph node dissection, recurrence and metastasis are given in Table 1. In AST (melanocytoma with spitzoid features) group, the age of the patients ranged from 3 to 57 years, with a mean age of 27.3 years. Thirteen patients (33.3%) out of 39 were under the age of 18, while 7 of them (17.9%) were in prepubertal period (<12 years). Thirty (77%) of the patients with AST were female, while 9 (23%) were male (female/male ratio: 3.33). Most of the lesions were localized in the extremities (69%), followed by the trunk (18%), head and neck (13%). At time of diagnosis, the size of the lesions ranged from 2 to 11 mm, mean of them 6.03 mm. Sentinel lymph node biopsy was performed in 4 patients (10.2%). Metastasis was not detected in any of them. One patient with AST underwent regional lymph node dissection and micrometastasis was detected in 1 of the 13 lymph nodes. This patient was a 41-year-old female and was alive after a follow-up period of 15 months (Figures 1a–1d). Follow-up clinical data were available for 20 out of 39 (51%) AST patients, ranging from 3 months to 84 months (mean 40.8 months, median 18 months). All the patients were alive without recurrent or metastatic disease at the time the data were collected for analysis. There were no follow-up data available in 19 AST patients.

**Figure 2:**
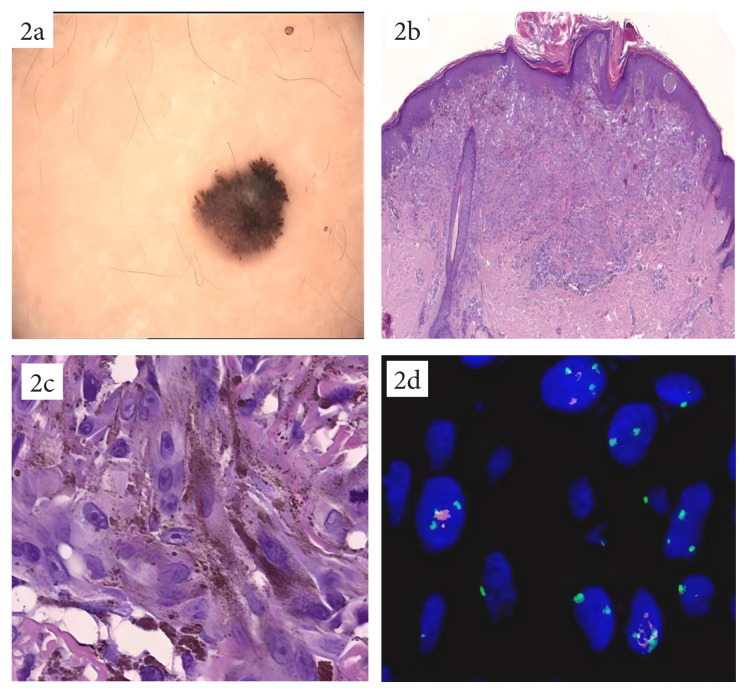
A case of prepubertal melanoma with spitzoid features. A heavily pigmented lesion with irregular borders on the right arm (a), a spitzoid lesion with hyperkeratotic and acanthotic epidermis, with relative symmetry (HEx40) (b), presence of many deep mitoses and uneven distribution of melanin pigment, reaching the deeper parts of the lesion (c), homozygous 9p21 deletion in 45% of tumor cells (9p21 FISH-positive) (d).

In the group of melanomas with spitzoid features, the patients’ age ranged from 6 to 56 years with a mean age 32.4 years (Figures 2a–2d). Two patients (20%) out of 10 were under the age of 18, while 1 (10%) was in prepubertal period (<12 years). Six (60%) of the patients were female, while 4 (40%) were male (female/male ratio: 1.5). The most common site was extremities (60%). At time of diagnosis, the size of the lesions ranged from 3 to 15 mm with a mean of them 6.4 mm. Sentinel lymph node biopsy was performed in 4 patients, none of the patients underwent lymph node dissection. Metastasis was not detected in any of them. The follow-up period ranged from 8 months to 84 months (mean 40 months, median 24 months) in 5 patients who were alive without recurrent or metastatic disease at the time the data were collected for analysis. No follow-up data were available for the remaining 5 patients.

### 3.2. Histopathological findings

Histopathological findings are summarized in Table 2. None of the parameters were diagnostic alone, but the sum of the features favored the diagnosis of either melanoma or AST as dictated by guidelines. As the features of both groups were compared, parameters such as asymmetry (p = 0.0047), loss of maturation (p = 0.035) and cytological atypia (p = 0.027) were found statistically significant in the differential diagnosis.

### 3.3. FISH analysis

The results of the 4-probe melanoma FISH and 9p21 FISH analyses are summarized in Table 3. In the AST group, 21 cases (54%) demonstrated isolated MYB deletion, seen in more than 40% of tumor cells, using melanoma FISH probes. One case displayed MYB deletion, together with RREB-1 gain, which was above the threshold level which is 29%. Out of 39, 12 cases (31%) showed homozygous 9p21 deletion. The percentage of cells with homozygous deletion in FISH-positive cases varied from 34% to 82%, with an average of 48%. Chromosomal alterations were demonstrated in 23 cases (59%) either using melanoma FISH or 9p21 FISH probes. Eleven cases with homozygous 9p21 deletion also had isolated MYB deletion.

In the group of melanomas with spitzoid features, using melanoma FISH probes, 8 cases out of 10 (80%), demonstrated the isolated MYB gene deletion, which was detected in more than 40% of the tumor cells. Six cases (60%) demonstrated homozygous 9p21 deletion. The percentage of cells with homozygous deletion in FISH-positive cases varied from 45% to 95%, with an average of 66%. Chromosomal alterations were demonstrated in 8 cases (80%), either using melanoma FISH or 9p21 FISH probes. In 6 cases, isolated MYB deletion, together with homozygous 9p21 deletion were detected.

Using conventional melanoma FISH probes, polyploidy, either as triploidy or tetraploidy, was detected in 9 cases (90%) in group of melanomas with spitzoid features and in 32 cases (82%) in AST group. Using 9p21 FISH probes, polyploidy was detected in 7 cases (70%) in the group of melanomas with spitzoid features and in 33 cases (85%) in AST group.

### 3.4. Comparison of FISH findings with the clinical and histopathological data

A comparative analysis was performed between 9p21 FISH and melanoma FISH results and the clinical and histopathological data.

Among the histopathological parameters, there was a statistically significant correlation between 9p21 FISH-positivity and deep mitoses (p = 0.029). In addition, we observed that epidermal consumption (p = 0.07) and increased mitotic activity (p = 0.05) were more frequent in cases with homozygous 9p21 deletion, but these differences did not reach statistical significance.

There was no statistically significant correlation between melanoma FISH assay and any of the histopathological or clinical data (Table 3).

## 4. Discussion

Currently, conventional light microscopic examination is the gold standard for diagnosis of melanocytic lesions [14]. However, routine light microscopic and additional immunohistochemical examination do not always provide adequate clinical information for certain lesions such as those with spitzoid features. This is primarily due to lack of consensus between the pathologists regarding the morphological findings and histopathological criteria [15]. Newer methodologies are essential for proper classification and diagnosis, regarding the last WHO classification of skin tumors, WHO 2018. Recent studies of the genomics of melanocytic tumors have shown that Spitz lineage tumors harbor HRAS mutation or kinase fusion. Accordingly, Spitz tumors are classified as Spitz nevus, atypical Spitz tumor (AST) or melanocytoma with Spitz lineage and Spitz melanoma. On the hand in conventional melanomas, resembling Spitz tumors or not, the initiating event is the involvement of BRAF, NRAS, KIT or NF1 mutations. Loss of CDKN2A has also been shown to have correlation with higher risk in Spitz tumors [16,17].

FISH is one of the techniques that would help to provide more information regarding the molecular pathways. As a result, a number of studies using conventional melanoma FISH probes (RREB1, CCND1, MYB, CEP6) have been performed in recent years. Gerami et al. declared that the sensitivity and specificity were calculated as 83% and 94%, respectively, in a series of 123 melanomas and 110 nevi, by using conventional melanoma FISH probes [18]. On the other hand, recent studies have shown that sensitivity values decrease in the spitzoid lesions using the conventional FISH probes. In addition, prognostic determination is very limited in tumors such as AST due to their low or indeterminate potential for malignancy. The sensitivity was reported as 43% and the specificity as 80% in a study using conventional melanoma FISH probes, in a series of 90 cases, half of which were spitzoid lesions [8, 19]. This has led a number of investigators to search for new methods to assess the correct diagnosis and prognosis.

Recently, Bastian et al. pointed out the deletions in Spitz tumors in the 9th chromosome in CGH [9]. Gerami et al. suggested that homozygous 9p21 deletion is a marker of aggressive disease in the spitzoid lesions [8,20]. Based on this data, Yazdan et al. suggested a risk classification that could be used for ASTs; i) spitzoid tumors showing homozygous 9p21 deletion (high risk), ii) spitzoid tumors showing 6p25 gene gain or 11q13 gene gain (medium-high risk), iii) spitzoid tumors showing 6q23 gene deletion (low risk), iv) spitzoid tumors with no FISH anomaly (low to very low risk) [21].

In this study, we tried to find out whether the use of conventional and more recent FISH probes, i.e. 4-probe FISH and 9p21 (CDKN2A) FISH, respectively, would aid the risk assessment in spitzoid lesions. We noted that abnormal FISH results were more common in melanomas with spitzoid morphology (either Spitz melanoma or spitzoid melanoma), compared to AST (60% compared to 30.8% for 9p21 FISH).

By conventional melanoma FISH probes, isolated MYB deletion (6q23 deletion) was the most common finding among AST and melanomas with spitzoid features (8 out of 10 in melanoma group, 21 out of 39 in AST group). In tumors with Spitz lineage, MYB (6q23) deletion pointed out low risk, as indicated by Yazdan et al. [21]. However, there was one case of AST, showing both MYB deletion and RREB-1 gain above the threshold levels. This finding arises a suspicion this tumor belongs to Spitz lineage or not. Unfortunately, we do not have the opportunity for NGS (next generation sequencing) in our series. This is a limitation for this study. However, it should be kept in mind that many pathology centers do not have access to this technology.

The aims of the study were to look for any clinical and histopathological parameter that correlated with FISH positivity. There was no statistically significant correlation between melanoma FISH assay and any of the histopathological or clinical data. On the other hand, among the clinical data, such as age, sex, localization, duration, size, and the clinical status, a significant correlation was observed between lesion diameter (size) and 9p21 FISH positivity. In a similar study, Busam et al. compared 9p21 FISH positivity with the clinical data in 75 patients with AST. In this series, in 6 out of 8 cases with homozygous deletion over threshold level, there was a strong correlation between FISH positivity and the factors related to the tumor progression, such as relapse, distant metastasis and disease related death (p = 0.001), These cases had a higher percentage of cells showing homozygous deletion, with an average of 80% [22]. In our study, the prognostic value of homozygous 9p21 deletion could not be demonstrated, although there were cases with high percentage of cells with homozygous deletion, much more above the cutoff threshold for a positive result, which is 29%. This may be due to the absence of or short follow-up period in certain patients, limiting our ability to provide more conclusive information regarding the prognostic value of these tests.

When we examined the histopathological features, such as symmetry, maturation, subcutaneous fat tissue involvement, ulceration, epidermal consumption, intraepidermal pagetoid spread, presence of Kamino bodies, cytological atypia and pleomorphism, expansive nodule, mitosis (number and location), atypical mitosis, and lymphocytic response, we were only able to show a statistically significant correlation between the 9p21 FISH-positivity and deep mitoses. Epidermal consumption and increased mitotic activity were more frequent in cases with homozygous 9p21 deletion, but these associations did not reach statistical significance. While not conclusive, these data may suggest that presence of deep mitoses, epidermal consumption and increased mitotic activity could be more worrisome features among the histopathological findings.

In conclusion, the histopathological distinction between benign and malignant spitzoid lesion primarily relies on the histopathological evaluation, as in other melanocytic lesions. There is no consensus regarding the incremental value of newly proposed ancillary techniques such as FISH probe analysis to improve the diagnostic accuracy amongst spitzoid lesions. Our data supports the notion that FISH probe analysis, together with both conventional 4-probe and 9p21 FISH, could aid the diagnostic accuracy in melanocytic spitzoid lesions such that the negativity of these assays strongly favors lower risk, since 9p21 FISH positivity was more common in melanomas with spitzoid features, compared to AST (60% compared to 30.8% for 9p21 FISH). But still there is no certainty. On the other hand, 9p21 FISH positivity correlated with more worrisome histopathologic and clinical features, such as deep mitosis, increased mitotic activity, epidermal consumption, and larger lesion size, so these features are precious, pointing out spitzoid lesions with higher risk. These data highlight the need for future studies using FISH or similar techniques with longer term follow-up in order to provide accurate prognostic information in lesions with spitzoid morphology.

## Figures and Tables

**Table 1:** The clinical features and the postdiagnostic parameters such as the status of sentinel lymph node biopsy, regional lymph node dissection, recurrence, and metastasis.

	Melanomas with spitzoid features n = 10	AST n = 39

**Age (years)**		
***-Mean***	**32.4**	**27.3**
***-Median***	**35**	**25**
***-Range***	**6–56**	**3–57**
***-Under 18 years***	**2 (20%)**	**13 (33.3%)**
***-Prepubertal (***≤***12 years)***	**1 (10%)**	**7 (17.9%)**

**Sex**		
***-Female***	**6 (60%)**	**30 (76.9%)**
***-Male***	**4 (40%)**	**9 (23.07%)**

**Site**		
***-Head-neck***	**2 (20%)**	**5 (12.8%)**
***-Trunk***	**2 (20%)**	**7 (17.9%)**
***-Extremities***	**6 (60%)**	**27 (69.2%)**

**Duration ** ***(month)***		
***-Mean***	**11.2**	**18.4**
***-Median***	**12**	**6**
***-Range***	**1–24**	**0.5–180**

**Size ** ***(mm)***		
***-Mean***	**6.4**	**6.03**
***-Median***	**7**	**6**
***-Range***	**3–15**	**2–11**

**SLNB after diagnosis**		
***-Performed***	**4 (40%)**	**4 (10.2%)**
• ***Negative***	**4 (40%)**	**4 (10.2%)**
• ***Positive***	**-**	**-**
***-Not performed***	**2 (20%)**	**17 (43.5%)**
***-Unknown***	**4 (40%)**	**18 (46.2%)**

**Regional lymph node metastasis**		
***-Performed***	**-**	**1 (2.6%)**
• ***Negative***	**-**	**-**
• ***Positive***	**-**	**1 (2.6%)**
***-Not performed***	**5 (50%)**	**20 (51.2%)**
***-Unknown***	**5 (50%)**	**18 (46.2%)**

**Recurrence**		
***-Yes***	**0 (0%)**	**0 (0%)**
***-No***	**5 (50%)**	
***-Unknown***	**5 (50%)**	

**Distant metastasis**		
***-Yes***	**0 (0%)**	**20 (51.2%)** **19 (48.7%)** **0 (0%)** **20 (51.2%)** **19 (48.7%)**
***-No***	**5 (50%)**	
***-Unknown***	**5 (50%)**	

**Follow-up (month)**		
***-Mean (month)***	**40**	**40.8**
***-Median(month)***	**24**	**36**
***-Range (month)***	**8–84**	**3–84**
***-Unknown***	**5**	**19**

**Clinical status**		
***-Alive without disease***	**5 (50%)**	**20 (51.2%)**
***-Unknown***	**5 (50%)**	**19 (48.7%)**

**Table 2:** The histopathological findings of the cases.

	Melanomas with spitzoid features n = 10	AST n = 39

**Asymmetry**	8 (80%)	12 (30.7%)

**Loss of maturation**	7 (70%)	13 (33.3%)

**Subcutaneous fat tissue involvement**	-	1 (2.56%)

**Ulceration/erosion**	1 (10%)	13 (33.3%)

**Epidermal ** ***consumption***	7 (70%)	21 (53.8%)

**Peripheral intraepidermal pagetoid spread**	2 (20%)	12 (30.7%)

**Kamino bodies**	-	11 (28.2%)

***Cytological atypia/pleomorphism***		
• ***Mild-moderate***	3 (30%)	26 (66.67%)
• ***Severe***	7 (70%)	12 (30.7%)

**Expansile nodules**	8 (80%)	21 (53.8%)

**Deep mitoses**	4 (40%)	6 (15.3%)

**Atypical mitosis**	2 (20%)	3 (7.7%)

***Lymphocytic response***		
•***None***	1 (10%)	5 (12.8%)
•***Nonbrisk***	7 (70%)	21 (53.8%)
•***Brisk***	2 (20%)	13 (33.3%)

**Table 3:** The results of FISH analysis.

	Melanomas with spitzoid features n = 10	AST n = 39
Melanoma FISH-positive	8 (80%)*	22 (56.4%)**
9p21 FISH-positive	6 (60%)	12 (30.8)
Chromosomal alterations detected by combination of both FISH analyses	8 (80%)	23 (58.9%)

* All cases showed isolated MYB deletion (6q23 deletion).

** Twenty-one cases showed isolated MYB deletion (6q23 deletion), one case had both MYB deletion and RREB-1 gain above the threshold levels.

**Table 4:** Comparison of FISH results and clinicopathological data.

	Melanoma FISH-positive* n = 30	9p21 FISH-positive n = 18	p values	

***Clinical parameters***			**Melanoma FISH-positive**	**9p21 FISH-positive**

• **Age**				
○ **Range**	3–56	3–45	p = 0.374	p = 0.292
○ **Under 18**	10 (33.3%)	7 (38.8%)		
○ **Prepubertal**	7 (23.3%)	6 (33.3%)		

• **Sex**				
○ **Male**	7 (23.3%)	4 (22.2%)	p = 0.308	p = 0.453
○ **Female**	23 (76.6%)	14 (77.7%)		

• **Site**				
○ **Head-neck**	5 (16.6%)	1 (5.55%)	p = 0.820	p = 0.381
○ **Trunk**	5 (16.6%)	4 (22.2%)		
○ **Extremities**	20 (66.6%)	13 (72.2%)		

• **Duration (month)**				
○ **Range**	0.5–180	0.5–24	p = 0.954	p = 0.938
○ **Mean**	18.69	10.2		

• **Diameter (mm)**				
○ **Mean**	6.16	6,8	p = 0.663	**p = 0.031**
○ **Median**	5.5	6.5		
○ **Range**	2–15	4–15		

• **Breslow thickness (mm)**				
○ **Mean**	2,255	1.887	p = 0.771	p = 0.293
○ **Median**	1.705	1.53		
○ **Range**	0.63–7.03	0.63–7.03		

***Histopathologic parameters***				

• **Asymmetry**	11 (36.6%)	6 (33.3%)	p = 0.990	p = 0.707

• **Lack of maturation**	15 (50%)	8 (44.4%)	p = 0.137	p = 0.598

• **Ulceration**	7 (23.3%)	6 (33.3%)	p = 0.308	p = 0.574

• **Epidermal consumption**	14 (46.6%)	13 (72.2%)	p = 0.782	p = 0.066

• **Peripheral pagetoid spread**	14 (46.6%)	8 (44.4%)	p = 0.965	p = 0.548

• **Kamino bodies**	6 (20%)	3 (16.6%)	p = 0.729	p = 0.324

• **Expansile nodule**	18 (60%)	16 (88.8%)	p = 0.884	p = 0.154

• **Mitoses**				
○ **2–6/mm2**	18 (60%)	15 (83.3%)	p = 0.422	**p = 0.05**
○ **>6/mm2**	6(20%)	2 (11.1%)		

• **Deep mitoses**	6 (20%)	6 (33.3%)	p = 0.775	**p = 0.029**

• **Atypical mitoses**	2 (6.66%)	2 (11.1%)	p = 0.363	p = 0.342

• **Brisk Iymphocyticresponse**	17 (56.6%)	9 (50%)	p = 0.908	p = 0.678

* All cases showed isolated MYB deletion (6q23 deletion), except one case with both MYB deletion and RREB-1 gain.
